# The Effect of High-Dose Postpartum Maternal Vitamin D Supplementation Alone Compared with Maternal Plus Infant Vitamin D Supplementation in Breastfeeding Infants in a High-Risk Population. A Randomized Controlled Trial

**DOI:** 10.3390/nu11071632

**Published:** 2019-07-17

**Authors:** Adekunle Dawodu, Khalil M. Salameh, Najah S. Al-Janahi, Abdulbari Bener, Naser Elkum

**Affiliations:** 1 Department of Pediatrics, College of Medicine, University of Cincinnati, Cincinnati, OH 45267, USA; 2 Division of Pediatrics, Al-Wakra Hospital, Hamad Medical Corporation, Doha, Qatar; 3 Department of Obstetrics and Gynecology, Women’s Hospital, Hamad Medical Corporation, Doha, Qatar; 4 Department of Biostatistics and Medical Informatics, Cerrahpasa Faculty of Medicine, Istanbul University Cerrahpasa and Istanbul Medipol University, 34098 Cerrahpasa-Istanbul, Turkey; 5 Sidra Medicine, Doha, Qatar

**Keywords:** vitamin D deficiency, supplementation, breastfeeding, mothers, infants

## Abstract

In view of continuing reports of high prevalence of severe vitamin D deficiency and low rate of infant vitamin D supplementation, an alternative strategy for prevention of vitamin D deficiency in infants warrants further study. The aim of this randomized controlled trial among 95 exclusively breastfeeding mother–infant pairs with high prevalence of vitamin D deficiency was to compare the effect of six-month post-partum vitamin D_3_ maternal supplementation of 6000 IU/day alone with maternal supplementation of 600 IU/day plus infant supplementation of 400 IU/day on the vitamin D status of breastfeeding infants in Doha, Qatar. Serum calcium, parathyroid hormone, maternal urine calcium/creatinine ratio and breast milk vitamin D content were measured. At baseline, the mean serum 25-hydroxyvitamin D (25(OH)D) of mothers on 6000 IU and 600 IU (35.1 vs. 35.7 nmol/L) and in their infants (31.9 vs. 29.6) respectively were low but similar. At the end of the six month supplementation, mothers on 6000 IU achieved higher serum 25(OH)D mean ± SD of 98 ± 35 nmol/L than 52 ± 20 nmol/L in mothers on 600 IU (*p* < 0.0001). Of mothers on 6000 IU, 96% achieved adequate serum 25(OH)D (≥50 nmol/L) compared with 52%in mothers on 600 IU (*p* < 0.0001). Infants of mothers on 600 IU and also supplemented with 400 IU vitamin D_3_ had slightly higher serum 25(OH)D than infants of mothers on 6000 IU alone (109 vs. 92 nmol/L, *p* = 0.03); however, similar percentage of infants in both groups achieved adequate serum 25(OH)D ≥50 nmol/L (91% vs. 89%, *p* = 0.75). Mothers on 6000 IU vitamin D_3_/day also had higher human milk vitamin D content. Safety measurements, including serum calcium and urine calcium/creatinine ratios in the mother and serum calcium levels in the infants were similar in both groups. Maternal 6000 IU/day vitamin D_3_ supplementation alone safely optimizes maternal vitamin D status, improves milk vitamin D to maintain adequate infant serum 25(OH)D. It thus provides an alternative option to prevent the burden of vitamin D deficiency in exclusively breastfeeding infants in high-risk populations and warrants further study of the effective dose.

## 1. Introduction

Chronic vitamin D deficiency resulting in rickets remains a serious childhood public health problem worldwide, particularly in breastfeeding infants who lack sun exposure and vitamin D supplementation in Asia, parts of Africa and the Middle East, including Qatar, and among the immigrant population from the above countries to Europe, Australia and New Zealand [1,2]. Although vitamin D is effective in preventing vitamin D deficiency, recent reports confirmed that nutritional rickets due to vitamin D deficiency is common and the prevalence is significantly higher in Middle Eastern countries [3], some parts of Asia [4] and among children of immigrant and minority populations than the Western population in prospective and cross-section studies [5]. Of 540 Qatari children of less than five years of age attending a primary health care clinic, 24% were found to have nutritional rickets [3]. A national survey of vitamin D deficiency in Mongolian children <5 years found that 42% of the children had serum 25-hydroxyvitamin D [25(OH)D] < 23 nmol/L and 50% of those with serum 25(OH)D < 23 nmol/L had clinical rickets [4]. Despite reports of high prevalence of nutritional rickets, especially in high risk populations worldwide [1,2,3,4,5], and the different recommendations from professional bodies and expert advisory groups on preventive measures [1,6], the prevalence of vitamin D deficiency associated with increased risk of rickets continues to be a significant public health problem, especially in breastfed infants [2,7,8,9] due to low vitamin D intake from the breast milk, low vitamin D supplementation and lack of sunlight exposure. The prevalence and magnitude of vitamin D deficiency depends on the definition used in the reported studies. The Institute of Medicine defines vitamin D deficiency as serum 25(OH)D < 30 nmol/L [9] while the Endocrine Society defines serum 25(OH)D < 50 nmol/L as vitamin D deficiency [10]. Some studies report the prevalence of 25(OH)D < 25 nmol/L as cutoff for vitamin D deficiency in unsupplemented breastfed infants with 43% in New Delhi, India [11], and 58% in a cohort of Arab breastfed infants in Doha, Qatar [12]. Furthermore, the prevalence of serum 25(OH)D < 30 nmol/L associated with increased risk of nutritional rickets [1] range from 13% in Cincinnati, Ohio [13], and 22% in Mexico City [13]. In a review of global vitamin D status based on articles published in PubMed/Medline, the prevalence of vitamin D deficiency as defined in the present study (serum 25(OH)D < 50 nmol/L) [10] in infants range from 24%, 40%, 46% to as high as 93%, 96% and 99% in Argentina, Australia, Black American (US), in Iran, Kuwait and India respectively [14].

Besides rickets, vitamin D deficiency or suboptimal vitamin D intake in infancy has also been shown to be associated with increased risk of lower respiratory tract infections in infancy and childhood [15,16] and vitamin D deficiency should, therefore, be regarded as a significant public health problem in children and prevention, particularly in the breastfed infants, requires heightened attention. The natural sources of vitamin D after birth in breastfeeding infants are previous transplacental transfer, human milk and sunlight exposure. The infant vitamin D stores at birth are dependent on maternal vitamin D status during pregnancy [17]. There are many documented reports of high prevalence of vitamin D deficiency during pregnancy worldwide associated with lack of sunlight exposure and inadequate vitamin D corrective supplements [8] that will predispose their infants to low vitamin D stores at birth [17]. The few studies that have also reported vitamin D status of breastfeeding mothers, indicate high prevalence of vitamin D deficiency (serum 25(OH)D < 50 nmol/L) ranging from 15% in Cincinnati [13], 51% in Shanghai [13], and 62% in Mexico City [13] which would theoretically be associated with low milk vitamin D intake for the nursing infant.

Since human milk contains low vitamin D (20–70 IU/L), it would be insufficient to meet the recommended daily intake of 400 IU of vitamin D for infants [6,9] when infant sun exposure is limited and the mothers are vitamin D deficient. Therefore, the current recommendation from professional body in the U.S. [6], and experts’ opinion [1,9,10] is that all infants receive direct oral supplementation of 400 IU vitamin D/day to maintain adequate vitamin D status to prevent vitamin D deficiency. Compliance with vitamin D supplementation in breastfeeding infants has been reported to be very low and recent studies from the US found vitamin D supplementation rate of 5%–19% in fully breastfed infants during the first six months of life [18,19]. In addition, vitamin D supplementation of breastfeeding infants does not address the concomitant high prevalence of vitamin D deficiency in their mothers. Therefore, ensuring maternal vitamin D sufficiency has been suggested as part of the strategy to prevent vitamin D deficiency in breastfed infants and their mothers [8,20]. This led to a pilot study of high-dose maternal vitamin D supplementation alone to prevent vitamin D deficiency in breastfeeding mother and her nursing infant.

Because of increasing reports of rickets [21] and the high prevalence of vitamin D deficiency in breastfeeding infants [6,8,21] and the new data in adults on the safety of up to 10,000 IU/day of vitamin D supplementation [22], a larger study from Charleston, South Carolina in the US, compared the effect of daily high-dose maternal vitamin D supplementation alone of 6400 IU with maternal supplementation of 400 IU/day and direct infant supplementation of 300 IU/day for six months on vitamin D status of breastfeeding infants [23]. The authors found significantly increased maternal serum 25(OH)D and breast milk vitamin D content in the maternal high-dose group [23]. The mean serum 25(OH)D in the nursing infants following high-dose maternal supplementation alone were similar to those in infants following maternal plus infant supplementation. There were no safety concerns related to vitamin D supplementation in mothers and infants as measured by serum calcium levels and urine calcium–creatinine ratio [23]. In a more recent large National Institute of Health-funded randomized controlled trial the same group from Charleston, South Carolina, compared the effectiveness of maternal vitamin D_3_ supplementation of 6400 IU/day alone with maternal plus infant supplementation with 400 IU/day in 334 exclusively breastfeeding mother–infant pairs. The authors confirmed that maternal supplementation alone with 6400 IU/day significantly increased maternal vitamin D status to meet the requirement of the nursing infant without safety issues [24]. The vitamin D content of the breast milk was not measured in this later study to assesses the contribution of milk vitamin D. In view of the reported high prevalence of vitamin D deficiency in Arab mother–infant breastfeeding dyads [8], reported low rate of infant vitamin D supplementation, and the possible effect of baseline vitamin D status on the response to supplementation dose [25,26], the present randomized controlled trial was conducted to compare the effect of high-dose maternal vitamin D supplementation alone with combined maternal and direct infant vitamin D supplementation on vitamin D status of breastfeeding infant in this high-risk population. To our knowledge, this was the first study to further examine and compare with previous landmark US studies the effect of daily maternal vitamin D supplementation alone on maternal and breast milk vitamin D status as part of a strategy to reduce the burden of vitamin D deficiency in breastfeeding mother–infant pairs in a population with endemic vitamin D deficiency.

### 1.1. Primary Aim

The effect of 6000 IU/day maternal vitamin D_3_ supplementation alone was compared with maternal supplementation of 600 IU/day plus direct infant supplementation of 400 IU/day vitamin D_3_ on the serum 25(OH)D levels of breastfeeding mothers and their infants including the percentage of mothers and infants that achieved a priori criteria of adequate serum 25(OH)D levels of ≥50 nmol/L [9].

### 1.2. Secondary Aim

The effect of maternal high-dose (6000 IU/day) vitamin D_3_ and 600 IU/day maternal vitamin D_3_ supplementation on human milk vitamin D content was also evaluated.

The hypothesis was that 6000 IU/day maternal vitamin D_3_ supplementation alone would optimize vitamin D status of exclusively breastfeeding mother and maintain vitamin D status of the nursing infant at equivalent level to that of an infant on direct oral vitamin D_3_ supplementation of 400 IU/day plus maternal 600 IU/day vitamin D_3_ supplementation.

## 2. Materials and Methods

### 2.1. Trial Design, Setting and Participants

This was a randomized, controlled, double-blind trial of the effect of 6000 IU/day maternal vitamin D_3_ supplementation alone versus maternal vitamin D_3_ supplementation of 600 IU/day plus direct infant vitamin D_3_ supplementation of 400 IU/day on the vitamin D status of breastfeeding infants in a sunny environment of Doha, Qatar. Arab breastfeeding mothers in Doha and Al-Wakra, Qatar, who delivered at term (37–42 weeks) at Al-Wakra Hospital, Hamad Medical Corporation (HMC) and planned to fully breastfeed their babies for the first 4–6 months postpartum were eligible for the study and were enrolled within four weeks of delivery and followed up for six months (during the period of August 2013–May 2016).

The study was approved by Hamad Medical Corporation and Weill Cornell Medical College Qatar Joint Institutional Review Board (JIRB No. 13-00036), and Cincinnati Children’s Hospital Medical Center Institutional Review Board (IRB) (study No. 2013–4909). Subjects were included in the study if they met the following criteria: (1) Arab women who delivered at term and presented for routine follow-up within 4 weeks after delivery, (2) were self-reported to be in good health, (3) agreed to blood and milk collection at enrollment and follow up to blood draw from the infant for study investigations, (4) planned to fully breastfeed for at least 4–6 months and (5) would be available for follow up visits. Exclusion criteria were mothers with pre-existing calcium disorders, active thyroid disease, Type 1 diabetes or liver diseases, which are likely to affect vitamin D status of the mothers and those of the infants. Mothers of infants with major or multiple congenital anomalies were also excluded.

### 2.2. Initial Visit and Baseline Data

Each mother completed questionnaires on socio-demographic and health status. These included maternal age, nationality, and educational level and occupation of herself and the father of the infant. Pregnancy and delivery information, and infant dietary and neonatal history including growth parameters were recorded. Maternal and infant sunlight exposure behaviors was based on usual outdoor clothing and included body surface area (BSA) exposed to sunlight, duration of sun exposure outdoor (h/week) and sun index score (%BSA x h/week of sun exposure), which correlate with serum vitamin D status in adults and infants [18,27]. The season in which the blood samples were drawn were defined as “hot season” (April–September) and “cool season” (October–March). Baseline biochemical parameters including serum vitamin D status and calcium homeostasis were assessed as a function of season and sunlight exposure characteristics at first visit. The data provided information with which to compare the results derived from different supplementation groups and at other different time points.

Maternal weight and height were recorded to determine the body mass index (BMI), (weight (kg)/height (meter squared)). BMI was not included as criterion for exclusion because of the high prevalence of overweight and obesity in the Arab population. It was taken into account in the analysis as possible confounding variable.

### 2.3. Interventions

#### Vitamin D Supplementation, Randomization, Blinding

The mother’s vitamin D tablets of 6000 IU or 600 IU were of similar color and taste and were manufactured and supplied by Tischon Corp (Salisbury, Maryland) as in our previous randomized controlled trial (RCT) [25] and each mother received a 100-day supply for three months visit and was repeated at the fourth visit. Biotics Research Corporation (Rosenberg, Texas, USA) manufactured and supplied infant vitamin D_3_ drops and the placebo administered daily to each infant. The medication met FDA guidelines and has been used in breastfeeding infants in the USA [24]. The vitamin D drops contained 0 IU (placebo) and 400 IU that were similar in taste, appearance and smell. Mothers were instructed to administer one drop daily to their nursing infants. The investigators, patients, and health care providers were blinded to treatment.

The onsite physician-investigators, assisted by research nurse coordinators, enrolled eligible patients after obtaining informed consent. The consented mother–infant pairs were randomly assigned to compare two treatment regimens of vitamin D_3_ supplementations: (a) high-dose maternal supplementation with 6000 IU/day of vitamin D_3_ and the infant received placebo and (b) maternal supplementation with 600 IU/day of vitamin D_3_ plus the infant receiving 400 IU/day of vitamin D_3_ orally. The literature suggests that 1000 IU vitamin D supplementation would increase milk vitamin D by 80 IU/L [23,28]; therefore, high-dose 6000 IU vitamin D_3_ maternal supplementation was chosen to improve maternal vitamin D status and increase milk vitamin D to a level that could meet the current need of the nursing infant. The current recommended lactating mother vitamin D intake of 600 IU and 400 IU infant intake [9] were chosen as control. The sampling procedure was designed by a statistician to achieve a seasonally balanced study population so that equal number of each group were enrolled during each of the two major seasons. A random assignment conducted as a stratified block design was computer generated to ensure equal number of mothers were randomly assigned to each of the two treatment groups monthly. Mothers on 6000 IU/day were provided with infant vitamin D drops with 0 IU vitamin D_3_, and mothers on 600 IU/day were provided with infant vitamin D drops with 400 IU vitamin D_3_. A secretary not involved in the project kept a list of randomization code.

### 2.4. Follow Up of Subjects

Research nurses assisted in the screening, enrollment, data collection and the follow up of mothers and the infants. The research coordinators completed the questionnaires and schedule appointments for blood draw for vitamin D and calcium homeostatic parameters, anthropometric measurement, pill count, as well as urine and breast milk collection. A computer generated calendar served as a reminder to contact the patients prior to their appointment. Each month, the study data manager and the research nurses generated electronic report on patient recruitment and retention, and every effort was made to reschedule patient for missed appointment.

### 2.5. Measurement of Outcome Variables

Maternal serum 25(OH)D, parathyroid hormone (PTH) and calcium were monitored at visit 1(enrollment) within 4 weeks postpartum, visit 4 (4 months postpartum after 3 months of vitamin D supplementation), and visit seven (seven months postpartum after six months of vitamin D supplementation). Infant serum 25(OH)D, PTH, and Ca, were also monitored at visits 1, 4 and 7. Serum calcium and maternal urine Ca/Cr ratio detects any possible episodes of hypercalcemia and hypercalciuria. The PTH versus vitamin D status relationship was included to compare response to vitamin D supplementation. Maternal urine pregnancy tests were performed monthly. If the mother was found to be pregnant, she would be informed and exit the study because of the uncertainty of the effect of giving high-dose maternal medication to the mother during the first trimester of pregnancy.

### 2.6. Laboratory Methods

Maternal and infant serum 25(OH)D and intact PTH. Total serum 25(OH)D levels were measured in HMC chemical laboratory by direct competitive chemiluminescence immunoassay on DiaSorin liaison platform (DiaSorin Liaison, Saluggia, VC, Italy). Serum intact PTH levels were measured also by chemiluminescence immunoassay (Unicel DxL 600, Beckman Coulter, Inc, CA, USA) as previously reported [12]. Vitamin D deficiency was defined a priori as serum 25(OH)D levels < 50 nmol/L [10] and vitamin D adequacy as serum 25(OH)D 50 nmol/L or greater [9] for this study.

Maternal and infant calcium measurement. The serum concentrations of calcium and urinary calcium and creatinine were measured in HMC Clinical Laboratory using standard analytic methods.

Breast milk vitamin D content. A 25 ml aliquot of full breast milk expression was collected at visits 1, 4 and 7 and frozen. The milk vitamin D content was measured by the use of LC-MS/MS Mass spectrometry techniques. Solvents were removed under a vacuum and samples were purified with the use of 2 chemically different HPLC systems. The final quantitation of the vitamin D and 25(OH)D was achieved by LC_MS/MS. The day-to-day inter-assay and intra-assay CVs for quantification were ≤ 12% [29]. The milk vitamin D contents were converted to antirachitic activity (ARA) using accepted conversion methods [29,30].

### 2.7. Safety Outcome

Each mother was monitored monthly for hypercalciuria. Vitamin D metabolites, serum calcium and urinary calcium/creatinine (Ca/Cr) ratio were monitored closely, and the results checked to detect any values in cautionary or higher than the predetermined parameters. Vitamin D_3_ supplementation was stopped if maternal serum calcium was >2.75 mmol/L and urine Ca/Cr ratio > 1 mmol/mmol [31]. Serum 25(OH)D > 95 ng/ml was defined as upper limit of serum 25(OH)D [10]. In recent studies with high-dose vitamin D supplementation of lactating women there were no evidence of toxicity as shown by either hypercalcemia or hypercalciuria [23,24]. Nonetheless, to ensure safety, Data Safety Monitoring Board (including chemical pathologist, endocrinologist, and a pediatrician) monitored reports of adverse events and compliance.

### 2.8. Sample Size Calculation

The primary outcome was infant serum 25(OH)D concentration at the seventh month postpartum and the secondary outcome was percentage of infants that achieved adequate vitamin D status defined a priori as serum 25(OH)D 50 nmol/L or greater. Based upon the data from published study [23], we calculated a percentage of infants in each group with serum 25(OH)D levels of at least 50 nmol/L (20 ng/ml) as successful outcome. Using these percentages as probability of a successful outcome, we estimated that the sample size of 160 mother–infant pairs will provide 90% power to show statistical non-inferiority (defined as <5% difference in success rate) in infants whose mothers alone were supplemented with vitamin D compared with infants whose mothers were supplemented with 600 IU and the infants also received oral supplementation and allowing for unexpected attrition rate which resulted in a total of 190 mother–infant pairs with 95 mother–infant pairs per group. This sample size also allowed us to compare maternal serum and milk vitamin D levels in between the groups over time using appropriate statistical analysis including linear mixed model.

### 2.9. Statistical Analysis

All patient demographic and outcome data were entered into a Red Cap database. The primary variables collected were maternal and infant serum 25(OH)D concentrations at visits 1, 4 and 7. Univariate statistics were generated to examine their distribution as well as missing values and potential outliers. We investigated the distribution of measured variables across groups to observe if there were any differential distribution problems. Kolmogorov Smirnov test, histogram, Q-Q plot, and box plot were used to control for normality. If variables were not normally distributed, they were presented as medians and differences examined by non-parametric tests. Student-t test was used to ascertain the significance of differences between mean values of two continuous variables and confirmed by non-parametric Mann–Whitney test. Spearman’s rank correlation coefficient was used to evaluate the strength of concordance between variables. A series of simple linear regressions were conducted to examine the contribution of each of the potential confounder or covariate variables on the outcome. Variables found to be significant in this analysis were included in the final model. The model was a general linear mixed model that incorporates the repeated nature of the data as well as potential confounder variables. Specifically, we used PROC and GENMOD with an identity link function and generalized estimating equation approach to model the relationship between vitamin D and the dose group while accounting for the potential confounding variables. We compared as secondary outcome the percentage of breastfeeding infants in the two groups that achieved serum 25(OH)D levels 50 nmol/L or greater at each time point in the study using Chi-squared test.

As secondary aim, analysis of variance was conducted to test the difference in terms of milk vitamin D content between the two maternal supplementation dose groups at the different time points. This was followed up with pairwise tests of the groups using Tukey’s post hoc tests to adjust for the potential multiple testing and control for other factors that may be associated with changes in vitamin D levels. Statistical analyses were performed using SAS 9.3 (SAS Institute Inc., Carry, NC, USA). The level *p* < 0.05 was considered as the cut-off value for significance.

## 3. Results

**Figure 1:**
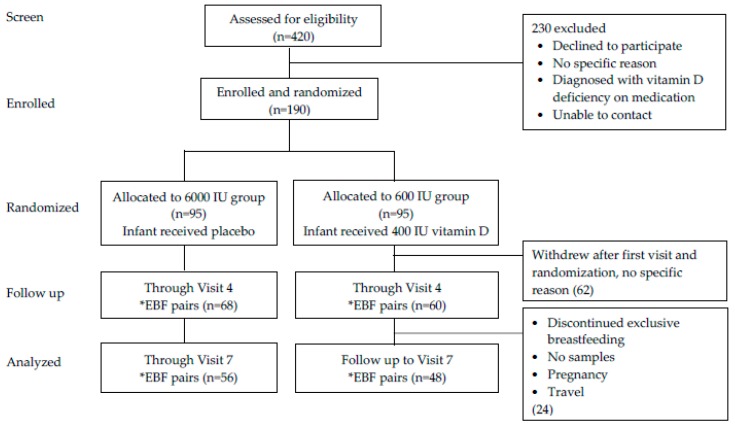
Flow chart of the participants throughout the study. *EBF—Exclusive Breastfeeding.

Of the 420 mothers that were assessed for eligibility, 190 consented to participate and were randomized with IRB acceptance to two treatment groups: maternal 6000 IU/day group plus infant placebo (0 IU/day vitamin D) versus maternal 600 IU/day group plus direct infant supplementation with 400 IU/day vitamin D_3_. Sixty-two (32%) of the mothers did not continue participation after randomization without any specific explanation but their baseline data were included in the analysis. One hundred twenty-eight mothers (67%) continued active participation through visit 4 while 104 (55%) completed the study to visit 7 for analysis (Figure 1).

The mean age of the mothers was 29.6 years, weight was 76 kg, and BMI 29.3. There were no significant differences in all the baseline characteristics including vitamin D status of the mothers and infants between the two groups and breast milk vitamin D content (Table 1 and Table 2). Of the mothers and infants with available data, 83% of the mothers were vitamin D deficient (serum 25(OH)D < 50 nmol/L) and 21% had serum 25(OH)D < 25 nmol/L, which is below serum 25(OH)D considered to be associated with osteomalacia [9]. Similarly, 82% of the infants were vitamin D deficient and 47% had serum 25(OH)D < 25 nmol/L, values which are associated with rickets [9]. The mean percentage of maternal body surface area (BSA) exposure outdoors of 13% with 0.32 hr/wk of sun exposure and mean sun index score of 6.6 were low. Although the infants BSA exposure outdoors (26%) was higher than in the mothers, the duration of sun exposure, 0.13 hr/wk, was lower. All the results indicated low maternal and infant sunlight exposure. On univariate analysis, maternal 25(OH)D correlated with BSA (rs = 0.2, *p* = 0.005) and infant 25(OH)D correlated with maternal BSA (rs = 0.19, *p* = 0.009) (Spearman’s correlation).

### 3.1. Follow Up

#### 3.1.1. Primary Outcome

The primary outcomes of the study were the serum 25(OH)D concentrations in both the mothers and the infants. Following intervention, maternal and infant serum 25(OH)D increased in both groups from baseline. The mean 25(OH)D concentrations were higher at visit 4 (88 nmol/L vs. 51 nmol/L, *p* < 0.0001) and at visit 7 (98 nmol/L vs. 51 nmol/L, *p* < 0.0001) in mothers on 6000 IU/day than those on 600 IU/day (Table 3). There was a higher serum 25(OH)D level in infants on direct vitamin D supplementation of 400 IU/day plus maternal supplementation of 600 IU/day than in infants of mothers on 6000 IU/day alone without infant supplementation (*p* < 0.001) at visit 4. At visit 7, serum 25(OH)D was slightly higher in infants on direct 400 IU/day vitamin D plus maternal 600 IU/day vitamin D supplementation compared with infants whose mothers alone were supplemented with 6000 IU/day vitamin D_3_ (*p* < 0.03) (Table 3).

#### 3.1.2. Secondary Outcome

The percentage of mothers and infants that achieved serum 25(OH)D ≥ 50 nmol/L, considered adequate by the Institute of Medicine based on the needs of 97.5% of the healthy population for bone health [9], were similar in the two groups at baseline; 18% in the 6000 IU group vs. 16% in the 600 IU group (*p* = 0.70). However, by visit 7, there was a significantly higher percentage of mothers in 6000 IU group with adequate vitamin D status than mothers in 600 IU group (96% vs. 52%, *p* < 0.0001) (Table 4).

For the infants, the percentage of infants with adequate vitamin D status (serum 25(OH)D ≥ 50 nmol/L) in the two groups were similar at all the visits and at visit 7, the end of study, (91% in infants on direct plus maternal supplementation vs. 89% in infants whose mothers alone were supplemented, *p* = 0.75) (Table 4).

#### 3.1.3. Serum PTH Findings

Serum PTH were evaluated to assess the PTH and vitamin D response to vitamin D supplementation during the intervention. At visit 7, the higher PTH in mothers in 600 IU group was associated with significantly lower serum 25(OH)D than those of mothers in 6000 IU group (51.3 vs. 39.7 pg/mL) (*p* < 0.003) (Table 5). There were no differences in the serum PTH levels of infants in the two groups at any of the visit times (Table 5).

#### 3.1.4. Breast Milk Vitamin D Content

**Figure 2:**
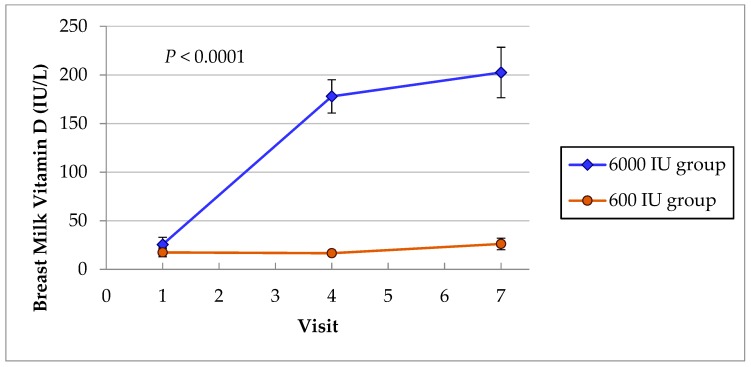
Breast milk vitamin D showed significant interactions between the groups. Mothers in 6000 IU group had substantial higher mean vD milk content of 202 IU/L compared with 26 IU/L in mothers in the 600 IU group at visit 7 (*p* < 0.0001).

The breast milk vitamin D content in mothers in the two groups was evaluated to compare the effect of maternal vitamin D_3_ supplementation of 6000 IU/day and 600 IU/day on milk vitamin D supply to their nursing infants. There was a very significant interaction between the milk vitamin D during the intervention (Figure 2). The mean vitamin D content at baseline were low in both groups of mothers (25 IU/L in 6000 IU group vs. 17.4 IU/L in 600 IU group, *p* = 0.36) (Table 1) and 57% of 182 lactating mothers had values below detection of the assay. After six months of vitamin D supplementation (visit 7), the mothers in 6000 IU group achieved higher mean vitamin D milk content of 202 ± 190 compared with 26.2 ± 38 in mothers in 600 IU group (*p* < 0.0001). The median values of milk vitamin D are compared in Table 6 using nonparametric tests since the data was not normally distributed.

#### 3.1.5. Safety Assessment

Total serum calcium levels were similar at baseline and at visits 4 and 7 in both groups of mothers. Similarly, the mean urine maternal calcium/creatinine ratios were similar at visits 1, 4 and 7 (Table 5) and there were no safety concerns. Serum calcium levels in the two groups of infants were also similar at visits 1, 4 and 7 (Table 5). The Data Safety Monitoring Board (DSMB) monitored the progress of the study and did not identify serious adverse events that warranted stopping the study.

#### 3.1.6. Health Outcomes

A. The mothers on 6000 IU (group 1) had a self-reported health status that was not significantly different than those on 600 IU (group 2) at visit 4 (*p* = 0.95) and at visit 7 (*p* = 0.83).

B. The health status reported for infants of mother in group 1 was not significantly different than those of mothers in group 2 at visit 4 (Fisher’s Exact test *p* = 0.83) and at visit 7 (*p* = 1.0).

C. Infant growth parameters, weight, length and head circumference, were also similar at baseline and on follow up between the two groups at visits 4 and 7 (Table 7).

## 4. Discussion

The high prevalence of vitamin D deficiency in the present study as early as one month postpartum justifies the need for a modified strategy for prevention of vitamin D deficiency in mother–infant dyads in Arab breastfeeding population possibly starting from pregnancy. The severity of vitamin D deficiency suggests that vitamin D deficiency in mothers and infants is an unrecognized public health problem [12] which is detrimental to mothers and their infants. 

Based on the definition of vitamin D deficiency, four out of five breastfed infants in this study had vitamin D deficiency at the first month of life, 47% had very low serum 25(OH)D level of <25 nmol/L, and over half (57%) had serum 25(OH)D lower than 30 nmol/L which is consistent with increased risk of rickets and lower respiratory infection without vitamin D supplementation or sunlight exposure [9,15,16]. The findings support the current reports that vitamin D deficiency in infants continues to be a significant global public health problem in low and middle resource countries worldwide [2,7] and warrants heightened attention. The explanations for the high prevalence of vitamin D deficiency in the exclusively breastfeeding infants in this study include lack of sunlight exposure shown by low mean sun index score of 4.52 compared with a mean sun index score of 68 in a cohort of breastfeeding infants in Cincinnati in summer [18], lack of vitamin D supplementation soon after birth due to “delay policy” of the Health Ministry of the country to supplement breastfeeding infants with vitamin D from age 2–12 months, and maternal vitamin D deficiency. Sunshine deprivation is common among mothers and infants in many Middle Eastern countries for cultural reasons and vitamin D supplementation rate of infants is low as in high income countries [18,19]. Since changes in sunlight exposure behavior may be difficult culturally, an alternative approach with high-dose maternal vitamin D supplementation alone was evaluated to optimize maternal vitamin D and milk vitamin D status to improve vitamin D nutrition of the nursing infant. Under research condition, the currently recommended direct supplementation of infants with 400 IU/day plus maternal supplementation of 600 IU achieved a slightly higher mean serum 25(OH)D of 109 nmol/L after 6 months of supplementation (visit 7) than 92 nmol/L in infants whose mothers alone were supplemented with 6000 IU/day and the infant received no supplementation (*p* < 0.03). Nonetheless, the percentage of infants in the two groups that achieved adequate serum 25(OH)D level of ≥50 nmol/L were similar (91% in infants on direct 400 IU/day plus maternal supplementation of 600 IU/day vs. 89% in infants of mothers in 6000 IU group and the infant received no supplementation (*p* = 0.75)) (Table 4). Furthermore, there were no differences in serum PTH response to vitamin D supplementation in the two groups at any of the visit times providing additional evidence of similarity in vitamin D status in response to vitamin D supplementation in both groups of infants (Table 5). Therefore, in the present study, maternal supplementation alone with 6000 IU/day vitamin D_3_ achieved adequate infant vitamin D status, similar to direct infant supplementation of 400 IU/day plus maternal supplementation of 600 IU/day vitamin D_3_.

A recent large randomized controlled trial indicated that serum 25(OH)D of infants who had direct vitamin D supplementation with 400 IU/day plus maternal supplementation of 400 IU/day and infants of mothers on 6400 IU/day supplementation alone and the infant received no supplementation were similar (108 vs. 98 nmol/L) [24] and our findings in infants are similar to theirs. It is also of interest that mean serum 25(OH)D of 90 nmol/L had been reported among unsupplemented infants of indigenous populations in East Africa who do not lack sunlight exposure [32], supporting a concept that “normal infant vitamin D concentration” could be naturally higher than is currently understood. The finding also suggests that vitamin D status could be “naturally higher” depending on sunshine and limited sun exposure in unsupplemented infants and be a significant contributor to infant vitamin D status.

Addressing maternal findings, the magnitude of vitamin D deficiency in the mothers was similar to those reported from India [11] but higher than 62% in breastfeeding mothers in Mexico City and 52% in mothers from Shanghai or 11% in a cohort of breastfeeding mothers in Cincinnati, USA [13,18]. In addition, the serum 25(OH)D levels of 35 nmol/L in the mothers were low compared with the baseline values of 82–90 nmol/L reported in breastfeeding mothers in a recent study from South Carolina, US [24]. Over one third of the mothers (36%) at baseline had serum 25(OH)D < 30 nmol/L which is consistent with increased risk of osteomalacia and non-skeletal disorders in adults [9,10] and is detrimental to the mothers, and vitamin D nutrition of their infants as referred to in earlier reports [23,24]. After 6 months of vitamin D supplementation, 6000 IU/day vitamin D_3_ supplementation was significantly more effective in optimizing maternal vitamin D status than 600 IU/day. Serum 25(OH)D increased by 62 nmol/L between visit 1 and visit 7 in mothers in 6000 IU group compared with only 17.5 nmol/L in mothers in 600 IU group (*p* < 0.0001). In addition, a higher proportion of mothers (96%) in 6000 IU group compared with 52% of mothers in 600 IU group achieved adequate vitamin D status at visit 7 defined a priori as serum 25(OH)D ≥ 50 nmol/L (*p* < 0.0001). PTH levels in mothers in 600 IU group was higher than in mothers in 6000 IU group probably due to significantly lower vitamin D status in 600 IU group following vitamin D supplementation (Table 3). In the randomized controlled trial from the U.S. [24], maternal supplementation alone with 6400 IU/day vitamin D_3_ also was more effective in elevating maternal serum 25(OH)D after 6 months of vitamin D supplementation than maternal supplementation with 400 IU/day. In that study there was a decline of 10.5 nmol/L in the 400 IU group but a 52 nmol/L increase after 6 months supplementation in the 6400 IU group (*p* < 0.0001). The increment in the serum 25(OH)D after six months of 6400 IU vitamin D supplementation is similar to the findings in this study. Therefore, the high-dose supplementation is more effective in optimizing maternal vitamin D status and the low dose is inadequate supplementation for high-risk, sunshine deprived population. It is of interest that at the end of the intervention 2% of mothers in the 6400 group and 13% in the 400 group would be considered vitamin D deficient (<50 nmol/L) in the US study [24]. In contrast, 4% of mothers in the 6000 group in the present study and almost half (48%) of those in the 600 group were considered vitamin D deficient. Part of the explanation for the difference in response to supplementation could be related to differences in baseline vitamin D status between mothers in this and the other study which has been shown to affect response to supplementation [25,26]. The mean 25(OH)D level of 98 nmol/L at the end of the study in 6000 IU group is still lower than mean 25(OH)D of 135 nmol/L reported at delivery among unsupplemented traditional female Tanzanian population in East Africa where mothers had no significant sunlight restriction as in breastfeeding mothers in Qatar [32]. Maintaining serum 25(OH)D ≥ 75 nmol/L has been associated with reduced risk of non-skeletal health disorders [10] and may be of theoretical benefit to a higher number of mothers in the 6000 IU group. If the objective of supplementation during lactation is to optimize vitamin D status in mother–infant pairs, then effective high-dose maternal supplementation still needs to be identified especially in high-risk population.

Monitoring of serum calcium, Ca/Cr ratio and serum 25(OH)D in our study did not show any significant difference between the two groups nor any serious adverse events that warrants stopping the study. Two infants had serum 25(OH)D of 94 ng/ml, below the predetermined upper limit of 25(OH)D without any other biochemical or clinical evidence of vitamin D toxicity. Safety parameters in two recent studies from South Carolina, US [23,24], which included serum calcium, and urine Ca/Cr ratios were similar in the two groups of mothers and infants, and revealed no evidence of vitamin D toxicity.

Although it is known that low human milk vitamin D content (range 5–80 IU/L) [24,33] contributes to the low vitamin D nutrition in unsupplemented breastfeeding infants who lack sun exposure, recent studies indicate that maternal vitamin D supplementation of 1000 IU/day would be expected to increase milk vitamin D content by 80 IU/L to improve vitamin D intake of nursing infants [23,28]. In the present study, baseline milk vitamin D levels were within the lower range of previously reported levels but 57% of the mothers in the two groups had undetectable vitamin D content and the milk provided no vitamin D for the nursing infant. After six months of vitamin D supplementation, the mean milk vitamin D level at visit 7 rose to a higher mean value of 202 IU/L in the 6000 IU group (Figure 2) or a median value of 143.7 IU/L (Table 6) than in the 600 group (*p* < 0.0001). The efficacy of the high-dose supplementation of 6000 IU to meet the current recommended infant vitamin D intake was lower than predicted. The lower efficacy may also be related to the effect of limited maternal sun exposure on breast milk vitamin D content as shown in another previous study [23]. It, however, supports our hypothesis that mothers in 6000 IU group would improve milk vitamin D, which would benefit her infant while mothers on 600 IU/day would only have marginal or lack milk vitamin D for their nursing infants. The mean vitamin D content of 202 IU/L in mothers in 6000 IU group was within the lower range of recommended vitamin D intake of 200–400 IU/day for nursing infants [24], while the milk vitamin D content of mothers in 600 IU group was very insufficient without direct vitamin D supplementation to meet the recommended daily intake of nursing infants, as shown in another study [23] in which milk vitamin D was measured. A recent large RCT which compared vitamin D status in infants of the mother who received 6400 IU/day alone with direct infant supplementation of 400 IU plus maternal supplementation of 400 IU/day also found similar vitamin D levels in the two groups of infants but the contribution of milk vitamin D was not compared because vitamin D content in the milk was not measured [24]. To the best of our knowledge, this would be the first study of the effect of high-dose maternal vitamin D supplementation on serum and milk vitamin D status as part of an alternative strategy to prevent vitamin D deficiency in infants in an Arab population with severe magnitude of vitamin D deficiency, and which provided comparison with results of previous landmark US studies [23,24]. Putting all the results together, 6000 IU/day maternal vitamin D supplementation alone appeared safe, optimizes maternal vitamin D status, increased milk vitamin D intake for nursing infants, and achieved similar adequate vitamin D status as in infants on direct 400 IU/day plus maternal 600 IU/day vitamin D supplementation.

There were no differences in the reported health status of the mothers and infants, and in the growth parameters of the infants between the two groups on follow up.

The limitations of our study include moderately adequate sample size, but a high dropout rate among the mothers mostly due to lack of compliance with exclusive breastfeeding and withdrawal from follow up with no specific reasons. Because the study is conducted in Arab breastfeeding mother and infant dyads, we only enrolled Arab mothers for this specific study. The results may be modified in other high-risk populations because of differences in sunlight exposure behavior and dose of vitamin D supplementation.

The present study provided additional valuable data to inform future research in other high-risk populations with similar population characteristics on the effective dose of daily high-dose maternal supplementation alone as an alternative option to reduce global burden of vitamin D deficiency in breastfeeding infants [24]. If successful, the strategy could provide data for health care providers to show that breast milk alone could be a source of vitamin D for breastfeeding infant with appropriate effective maternal high-dose vitamin D supplementation alone (6000–6400 IU or more). This option to prevent vitamin D deficiency in the breastfeeding mother–infant dyads would warrant public health education of health care professionals and providers. It is of interest that a recent study [34] found maternal preference for taking medication themselves as opposed to giving the vitamin D supplement to their infants. The authors suggested that maternal supplementation alone may improve vitamin D intake of the nursing infant in an environment where infant supplementation rate is low.

## 5. Conclusions

Maternal vitamin D_3_ supplementation alone with 6000 IU/day achieved similar adequate vitamin D status as in infants on direct vitamin D_3_ supplementation of 400 IU/day plus maternal 600 IU/day supplementation, and safely optimizes maternal vitamin D status with increase in milk vitamin D content. It supports high-dose maternal vitamin D supplementation alone as a possible alternative strategy to reduce the burden of global vitamin D deficiency in breastfeeding infants and their mothers and warrants more studies on appropriate effective dose especially in high-risk populations.

## Figures and Tables

**Table 1:** Baseline (Visit 1) characteristics and vitamin D status of exclusively breastfeeding mothers by maternal supplementation group.

Variables	N^++^	6000 IU Group (*n* = 95) Mean ± SD	N^++^	600 IU Group (*n* = 95) Mean ± SD	*p*-value
Age (years)	95	29.7 ± 5.0	95	29.5 ± 4.6	0.87
Weight (Kg)	95	77.4 ± 16.4	95	75.4 ± 16.4	0.40
Body Mass Index	95	29.4 ± 5.6	95	29.1 ± 6.0	0.33
Education	95		95		
• None/elementary		4		3	0.76
• High school		17		14	
• College/University		74		78	
Subjective health score	95	7.6		7.6	0.95
Season at enrollment (hot) %	95	50.5	95	51.6	0.88
Sun exposure behavior	95		95		
• % BSA exposure outdoors		11.7 ± 7.2		13.6 ± 12.1	0.19
• Sun exposure (h/week)		0.27 ± 0.88		0.38 ± 1.55	0.56
• Sun index score (% BSA x sun exposure h/week)		4.5 ± 15.9		8.7 ± 34.9	0.29
Serum Ca (mmol/L)	95	2.36 ± 0.08	94	2.34 ± 0.07	0.11
Urine CA/Cr ratio (mmol/mmol)	90	0.17 ± 0.15	90	0.14 ± 0.12	0.24
Serum 25(OH)D (nmol/L)	94	35.1 ± 16.3	94	35.7 ± 13.6	0.76
Serum PTH (pg/ml)	94	46.4 ± 25.7	93	50.4 ± 25.2	0.29
Breast milk vitamin D (IU/L)	92	25.5 ± 72.0	90	17.4 ± 42.4	0.36

++ N = number of observations. No significant differences between the two groups. PTH = parathyroid hormone.

**Table 2:** Baseline (visit 1) characteristics and vitamin D status of exclusively breastfeeding infants by maternal supplementation group.

Variables	N^++^	6000 IU Group (*n* = 95) Mean ± SD	N^++^	600 IU Group (*n* = 95) Mean ± SD	*p*-value
Weight (g)	95	3404 ± 509	95	3338 ± 448	0.34
Length (cm)	95	50.8 ± 2.9	95	50.8 ± 2.1	0.86
Head circumference (cm)	95	34.8 ± 2.2	95	34.7 ± 1.4	0.58
Sun exposure behavior					
• % BSA exposure outdoors	95	25.6 ± 13.2	95	26.3 ± 16.8	0.75
• Sun exposure (h/week)	95	0.15 ± 0.5	95	0.11 ± 0.39	0.55
• Sun index score (% BSA x sun exposure h/week)	95	5.1 ± 18.1	95	3.95 ± 14.7	0.63
Serum Ca (mmol/L)	95	2.70 ± 0.08	94	2.69 ± 0.09	0.79
Serum 25(OH)D (nmol/L)	93	31.9 ± 21.7	94	29.6 ± 16.1	0.41
Serum PTH (pg/ml)	91	30.4 ± 21.4	91	31.1 ± 21.8	0.84

++ N = number of observations. No significant differences were observed between the two groups.

**Table 3:** Comparison of serum 25(OH)D concentrations in exclusively breastfeeding mothers and infants by group and visit.

Variables	Visit	N^++^	6000 IU Group (*n* = 95) Mean ± SD	N^++^	600 IU Group (*n* = 95) Mean ± SD	*p*-value
Maternal Serum 25(OH)D nmol/L	1	94	35.1 ± 16.3	94	35.7 ± 13.6	0.76
	4	68	88.3 ± 32.2	60	51.4 ± 15.7	<0.0001 *
	7	56	98.2 ± 36.5	48	51.7 ± 19.8	<0.0001 *
Infant Serum 25(OH)D nmol/L	1	93	31.9 ± 21.7	94	29.6 ± 16.1	0.41
	4	67	81.4 ± 26.5	60	105.5 ± 50.4	0.001 *
	7	55	92.2 ± 35.5	47	109.1 ± 43.3	0.03 *

++ N = number of observations. * Maternal serum 25(OH)D (nmol/l) and infant serum 25(OH)D (nmol/L) were significantly different between the two groups at visit 4 and visit 7 (Two-sided T-tests).

**Table 4:** Categories of maternal and infant serum 25(OH)D status by visit and group.

Variables	Visit	6000 IU Group [n/N (%)]	600 IU Group [n/N (%)]	*p*-value
Maternal Serum 25(OH)D ≥ 50 nmol/L	1	17/94 (18%)	15/94 (16%)	0.70
	4	64/68 (94%)	30/60 (50%)	<0.0001 *
	7	54/56 (96%)	25/48 (52%)	<0.0001 *
Infant Serum 25(OH)D ≥ 50 nmol/L	1	18/93 (19%)	15/94 (16%)	0.54
	4	59/67 (88%)	53/60 (88%)	0.96
	7	49/55 (89%)	43/47 (91%)	0.75

*n* = number of observations with serum 25(OH)D value ≥ 50 nmol/L within the group and N is number of observations in each group. (%) is the percentage of serum 25(OH)D ≥ 50 nmol/L within each group. * Significant difference between the two groups of mothers at visits 4 and 7 (Chi-squared test).

**Table 5:** Comparison of maternal and infant serum calcium, PTH and maternal urine Ca/Cr ratio and infant serum calcium and PTH by group and visit.

Variables	Visit	N^++^	6000 IU Group (*n* = 95) Mean ± SD	N^++^	600 IU Group (*n* = 95) Mean ± SD	*p*-value
Maternal						
Serum Ca (mmol/L)	1	95	3.36 ± 0.08	94	2.33 ± 0.07	0.11
	4	68	2.35 ± 0.08	60	2.33 ± 0.08	0.26
	7	58	2.34 ± 0.08	47	2.34 ± 0.09	0.86
Serum PTH (pg/ml)	1	94	46.4 ± 25.7	93	50.4 ± 25.1	0.29
	4	68	40.1 ± 22.3	61	47.0 ± 20.1	0.06
	7	58	39.7 ± 18.1	48	51.3 ± 25.6	0.003 *
Urine Ca/Cr ratio (mmol/mmol)	1	90	0.16 ± 0.15	90	0.14 ± 12.0	0.24
	4	66	0.24 ± 0.27	60	0.20 ± 0.15	0.24
	7	56	0.24 ± 0.20	49	0.19 ± 0.13	0.13
Infant						
Serum Ca (mmol/L)	1	95	2.69 ± 0.08	94	2.69 ± 0.09	0.31
	4	67	2.62 ± 0.09	60	2.64 ± 0.09	0.31
	7	57	2.57 ± 0.09	48	2.54 ± 0.09	0.15
Serum PTH (pg/ml)	1	91	30.4 ± 21.4	91	31.1 ± 21.8	0.84
	4	64	20.7 ± 12.0	59	21.3 ± 13.0	0.78
	7	57	26.7 ± 28.0	47	26.7 ± 13.2	0.98

++ N = number of observations. * The maternal serum PTH significantly different between the two groups (Two-sided T-tests).

**Table 6:** Comparison of breast milk vitamin D between groups at Visit 1, Visit 4, Visit 7.

Variables	Visit	N^++^	6000 IU Group (*n* = 95) Median (Range)	N^++^	600 IU Group (*n* = 95) Median (Range)	*p*-value
Breast Milk Vitamin D (IU/L)	1	92	8.1 (8.1, 532.0)	90	8.1 (8.1, 379.9)	0.7657
	4	68	185.9 (8.1, 644.0)	60	12.1 (8.1, 70.8)	<0.0001 *
	7	54	143.7 (8.1, 852.5)	41	14.3 (8.1, 203.1)	<0.0001 *

++ N = number of observations. * The median milk vitamin D content were significantly different between the two groups at visits 4 and 7 (Wilcoxon Mann-Whitney Test).

**Table 7:** Comparison of Infant Birth Weight (g), Length (cm), and Head Circumference (cm) by Visit and Group.

Variables	Visit	N^++^	6000 IU Group (*n* = 95) Mean ± SD	N^++^	600 IU Group (*n* = 95) Mean ± SD	*p*-value
Weight (g)	1	95	3404.7 ± 509.3	95	3338.6 ± 448.4	0.34
Length (cm)		95	50.8 ± 2.9	95	50.9 ± 2.1	0.86
Head circumference (cm)		95	34.8 ± 2.2	95	34.7 ± 1.4	0.58
Weight (g)	4	68	7250.9 ± 849.3	62	7021.2 ± 783	0.11
Length (cm)		68	64.3 ± 2.5	62	64.1 ± 2.4	0.72
Head circumference (cm)		68	41.9 ± 1.5	62	41.6 ± 1.1	0.20
Weight (g)	7	58	8917.0 ± 1085.7	49	8789.0 ± 915.3	0.50
Length (cm)		58	70.7 ± 2.8	49	70.8 ± 3.1	0.84
Head circumference (cm)		58	44.8 ± 1.5	49	44.6 ± 1.0	0.32

++ N = number of observations. There were no significant differences between the two groups.
